# Rapid Estimation of Poly(3-hydroxybutyrate-co-3-hydroxyvalerate) Composition Using ATR-FTIR

**DOI:** 10.3390/polym15204127

**Published:** 2023-10-18

**Authors:** Sara Alfano, Francesca Pagnanelli, Andrea Martinelli

**Affiliations:** Department of Chemistry, Sapienza University of Rome, P.le Aldo Moro 5, 00185 Rome, Italy; sara.alfano@uniroma1.it (S.A.); francesca.pagnanelli@uniroma1.it (F.P.)

**Keywords:** poly(3-hydroxybutyrate-co-3-hydroxyvalerate), attenuated total reflection FTIR, composition determination, inverse linear regression

## Abstract

A great research effort is involved in polyhydroxyalkanoates (PHAs) production and characterization since they are an attractive degradable polyester family that potentially could substitute oil-based polymers. This is due to two main key factors: their production is sustainable, being that they are produced by microorganisms possibly fed by organic waste-derived products, and they are degradable. Moreover, PHAs’ thermal and mechanical properties could be tuned by varying their monomeric composition through the proper selection of microorganism feedstock and bioreactor operative conditions. Hence, a rapid and facile determination of the PHA chemical structure by widely available instrumentation is useful. As an alternative to the standard gas-chromatographic method, a new procedure for the composition determination of poly(3-hydroxybutyrate-co-3-hydroxyvalerate) (P3HBV), the most common PHA copolymer, by attenuated total reflection FTIR (ATR-FTIR) is presented. It is based on the linear dependence of selected and normalized absorption band intensity with the molar fraction of repeating units. To break free from the crystallinity variability, which affects the result reproducibility and data scattering, the polymer sample was rapidly quenched from the melt directly on the surface of the ATR internal reflection element and analyzed. The data obtained from 14 samples with a molar fraction of 3-hydroxybutyrate repeating units (*X_3HB_*) ranging from 0.15 to 1 were analyzed. According to preliminary analyses, the normalized intensity of two absorption bands was selected to develop a calibration method able to predict *X_3HB_* of unknown samples and to evaluate the related uncertainty through prediction intervals of inverse regression. The proposed method proves to be useful for an easy and rapid estimation of P3HBV composition.

## 1. Introduction

Polyhydroxyalkanoates (PHAs) belong to a wide family of polyesters produced by various microorganisms as an energy storage mechanism, triggered by alternating periods of feast and famine when the cells are fed intermittently [1]. After accumulation, PHAs can be extracted from biomass using different procedures [2,3,4]. The growing interest in PHAs is evident from the extensive research dedicated to them, driven by factors related to the production process and to the properties of the polymers. However, despite the progress, the production of PHAs is not yet as economically competitive as their petroleum-based polymer counterparts. Therefore, research efforts are focused on designing laboratory and pilot-scale plants capable of using mixed microbial cultures, cost-effective raw materials, and sustainable extraction methods to reduce the environmental and economic costs of PHAs. Nevertheless, PHAs possess key properties that make them highly appealing and enable them to compete with commodity polymers, such as polyethylene and polypropylene, in certain applications. Because of their natural origin, they are biodegradable and compostable and have a low vapor permeability, making them suitable for the packaging industry [5,6,7]. They are also biocompatible and are used in biomedical applications [8,9,10]. Furthermore, volatile fatty acid (VFA)-rich streams from the acidogenic fermentation of organic waste fractions, including municipal solid waste, urban wastewater treatment sludge, garden and park waste as well as selected waste from food processing, can be used as microorganism feedstock and makes the PHA production process part of a virtuous biorefinery cycle [11,12,13].

Another appealing feature of PHAs concerns the possibility to change their chemical structure regulating the substrate composition, mainly, and varying the production operating conditions, such as pH, temperature, and feeding cycle length [14,15]. Consequently, homopolymers, random and block copolymers composed of different repeating units, can be obtained by the careful design and control of the production process, leading to a wide range of structural, thermal, and mechanical PHA properties.

The prototypal PHA is poly(3-hydroxybutyrate) (P3HB), a homopolymer primarily obtained by using acetate as a carbon source for microorganism feeding. However, the high crystallinity, the brittleness, and a melting point close to its decomposition temperature limit its processability and use [16]. On the other hand, when microorganisms are fed with a mixture of C2–C5 volatile fatty acids, synthetic or coming from the controlled acidogenic fermentation of organic waste, poly(3-hydroxybutyrate-co-3-hydroxyvalerate) copolymer (P3HBV) is obtained. Since acetic and butyric acids are used for 3-hydroxybutyrate repeating units (3HB), whereas propionic and valeric acids are used for 3-hydroxyvaleate (3HV) comonomers, it is possible to have copolymers characterized by different composition and, hence, by different chemical, thermal, and mechanical properties [14,17,18]. For instance, a decrease in the P3HBV melting point and crystallinity can be reached by increasing the 3HV content up to around 40 mol%. This makes the copolymers more easily processable at lower temperatures and increases the biodegradation rate, the strain at break and toughness. Conversely, higher 3HV concentrations result in the opposite trend [19,20,21].

In addition, the composition of the extracted polyhydroxyalkanoate (PHA) copolymer can be affected by the extraction method and conditions, especially when poor solvents are used as alternatives to unsafe chlorinated solvents. This is because copolymers with different compositions have different solubilities, which can lead to fractionation during extraction [4].

Therefore, the knowledge of the copolymer composition is of utmost importance to control the production process operating conditions and predict the copolymer features required for specific applications.

Nowadays, there are two main methods for determining the P3HBV composition: ^1^H-NMR and gas chromatography (GC) [22]. However, the absolute ^1^H-NMR method is not used routinely due to the obvious reasons of instrument availability and cost, even though it provides the most precise results. On the other hand, GC is the most commonly used method, despite it being somewhat laborious and requiring calibration and instrumentation. This method was initially proposed by G. Braunegg et al., in 1978 [23], and has undergone minor variations since then [22]. It is based on the methanolysis of PHAs in the presence of sulfuric acid followed by the GC determination of the obtained monomer methylic ester. The relative abundance of 3HV and 3HB comonomers is calculated using a standard P3HBV of known composition. The proposed procedure offers the advantage of allowing direct analysis without the need for the prior extraction of polymers from the cells and the quantification of the total amount of polymer in the biomass. Then, the GC method is routinely used to characterize the extracted P3HBV composition and purity. 

In the 1986, Bloemberg et al. have suggested an alternative method based on FTIR spectroscopy [24]. The study reported a good correlation between the composition of P3HBV copolymers, as determined with ^1^H-NMR, and FTIR absorption band intensities. In particular, it has been observed that the ratio between the integrate absorbance of the C–H stretching region (3130–2770 cm^−1^) and the C=O stretching region (2000–1580 cm^−1^) is linearly related to the 3HV molar fraction. Meanwhile the relationship holds well with the FTIR results acquired from a chloroform polymer solution, it is linked to polymer crystallinity when the spectra were recorded from a P3HBV solid film casted on KBr pellets. The authors have argued that the composition of solid copolymer can be evaluated if the samples have quite similar levels of crystallinity. Specifically, this rare feature has resulted from the same isolation process carried out by solution precipitation of the P3HBVs. Actually, PHA spectra, as for other semi-crystalline polymers, are closely related to crystallinity, which brings about band shift, sharpening, and intensity variations as well as the appearance of bands of regular helical conformation [25,26,27].

Another alternative method to determine PHA composition has been proposed by Jos et al. [28] who used Raman spectroscopy and relied on the linear relationship between the ratio of the band integrals at 844 cm^−1^ (ν C-COO) to that at 1101 cm^−1^ (ν_s_ C-O-C, ρCH3) and the 3HV molar fraction. However, the model has been tested within a limited composition range (0 ≤ 3HV mol% ≤ 12), and the coefficient of determination obtained (R^2^ = 0.90) was relatively low. 

Stimulated by these studies and the evidence that FTIR instruments are more common in industrial laboratories and more easily accessible in academic laboratories than GC or Raman apparatus, as well as the frequent need for rapid analysis, this paper describes a new procedure to evaluate P3HBV composition based on infrared spectroscopy. In particular, the intensity of the selected absorption bands was related to the copolymer composition. Furthermore, to circumvent the unpredictable crystallinity of pristine real samples, attenuated total reflection FTIR (ATR-FTIR) spectra were obtained from samples immediately quenched from the melt onto the internal reflection element surface of the ATR device at room temperature. The data analysis demonstrates that the proposed method has proven effective for the rapid and easily accessible preliminary quantification of P3HBV composition.

## 2. Materials and Methods

### 2.1. Materials

The opportunity afforded to our research group for conducting physical–chemical characterizations on numerous P3HBV samples, extracted from biomasses obtained through various polymer production and extraction processes, has yielded a substantial collection of copolymers spanning a wide and representative composition range. All the analyzed samples have been obtained using soxhlet extraction with chloroform to reach high recovery yields and purities. However, any other extraction procedures that ensure sufficiently low impurity content that does not affect the PHA FTIR spectrum can be used. The samples were implemented with commercial P3HB (Biomer^®^ powder, Krailling, Germany). The samples were in the form of films, powders, or pellets.

### 2.2. Sample Preparation and Characterization

The copolymer composition, expressed as a molar fraction of 3-hydroxybutyrate repeating units (*X_3HB_*), were determined using the GC method, described elsewhere [23,29]. Briefly, approximately 3 mg of extracted PHA were suspended in 2 mL of acidified methanol solution (at 3% *v*/*v* H_2_SO_4_) containing benzoic acid (at 0.005% *w*/*v*), as an internal standard, and 1 mL of chloroform in a screw-capped test tube. Then, an acid of catalyzed methanolysis of polymer took place, and the formed methyl esters were quantified using gas-chromatography (GC-FID Perkin Elmer 8410). The relative abundance of 3HB and 3HV comonomers was determined using a commercial P3HBV copolymer with a 3HV content of 5 wt% (Sigma-Aldrich, Milan, Italy) as a reference standard. A maximum error in the monomeric unit concentration of 0.02 mol mol^−1^ was evaluated with repeated GC experiments. In this research, 14 different PHAs with *X_3HB_* spanning from 0.15 mol mol^−1^ to 1 mol mol^−1^ were analyzed. The adopted sample code is P3HBV-X, where X is the 3HB molar fraction in the copolymers.

The ATR-FTIR spectra were acquired using the Nicolet 6700 instrument (Thermo Fisher Scientific, Waltham, MA, USA) by co-adding 200 scans at a resolution of 4 cm^−1^. The ATR device is a Golden Gate (Specac, Orpington, UK) endowed with a diamond single internal reflection element (IRE) at 45°. The polymer samples were melted over a Kapton foil (about 1 cm × 1 cm) at 180 °C by using a Linkam HFS 91 hot stage (Linkam) driven by a Linkam TP 92 temperature controller. Alternatively, a heating plate (ArgoLAb M3-D, Carpi, MO, Italy) set at about 200 °C was used for some samples randomly selected. In both experiments, the molten samples were rapidly transferred on the IRE surface and the spectrum was collected in 30 s, according to adopted conditions. P3HBV-0.15, P3HBV-0.60, and P3HBV-0.92 samples were kept on the IRE surface further for 1 h at 25 °C. During this period, the cold crystallization occurred, allowing for the acquisition of polymer spectra in the semi-crystalline phase.

For each sample at different compositions, at least two independent experiments comprising quenching, spectrum acquisition, and elaboration were carried out. No variation of the spectra was found by melting the polymer on heating plates and hot stage, and nor was a clear indication of possible oxidation phenomena evidenced. Spectra elaborations, performed using Omnic software (version 1.70, Thermo Fisher Scientific, Waltham, MA, USA), comprised a baseline correction between 1510 cm^−1^ and 848 cm^−1^. The wavelengths were carefully selected in correspondence of spectrum valleys which showed no or negligible variation according to sample composition. Then, the intensities of the selected bands at 968 cm^−1^, 1004 cm^−1^, 1050 cm cm^−1^, and 1084 cm^−1^ where normalized with respect to the intensity of the band 1453 cm^−1^, used as an internal standard. The absorbance ratios were named $R_{\bar{\upsilon }}$, where $\bar{\upsilon }$ is the wavelength of the absorption band.

### 2.3. Data Analysis

Experimental data reported as absorption ratios (*y*) vs. *X_3HB_* (*x*) have been compared using statistical discrimination evaluating the confidence intervals for model parameters, the adequacy of linear models, and the prediction intervals for inverse regression. Confidence intervals on parameters were evaluated according to usual formulas [30].

(1) $${\hat{\beta }}_{0}\pm t_{\alpha /2,m-2}\sqrt{MS_{E}\left(\frac{1}{m}+\frac{{\bar{x}}^{2}}{S_{xx}}\right)}$$

(2) $${\hat{\beta }}_{1}\pm t_{\alpha /2,m-2}\sqrt{MS_{E}/S_{xx}}$$

where ${\hat{\beta }}_{0}$ is the estimated value of the intercept, ${\hat{\beta }}_{1}$ is the estimated value of the slope, $t_{\alpha /2}$ is the value of the *t* distribution corresponding to *α* = 0.05 and *m* − 2 degree of freedom and where *m* is the number of points used for calibration, *MS_E_* is the sum of square due to random errors evaluated in regression analysis, $\bar{x}$ is the mean of *x* values used in the calibration phase, and *S_xx_* is the sum of squares of *x* values used in the calibration phase.

Adequacy of the linear models was assessed using regression analysis and the lack of fit (LOF) test estimating the pure error variance (*PE*) by replicated measurements of *y* at fixed *x* and comparing it with the *MS_LOF_* term by an *F* test of hypotheses [30].

Prediction intervals for inverse regression (i.e., confidence intervals on the predicted *x* by measuring the *y* variable) were estimated considering two replicates of the *y* measurement according to the following formula [31]:

(3) $$\hat{x_{h}}\pm t_{\frac{\alpha }{2},m-2}\cdot \sqrt{S_{xh}^{2}}$$

with

(4) $$S_{xh}^{2}=\frac{{MS}_{E}}{{\hat{\beta_{1}}}^{2}}\cdot \left(1/n+\frac{1}{m}+\frac{{\left(\hat{x_{h}}-\bar{x}\right)}^{2}}{S_{xx}}\right)$$

where ${\hat{x}}_{h}$ is the *x* predicted value by inverse regression for a measured *y_h_* value replicated two times and *n* is the number of replicated *y* in the measurement phase.

## 3. Results

### 3.1. ATR-FTIR Analysis

**Figure 1:**
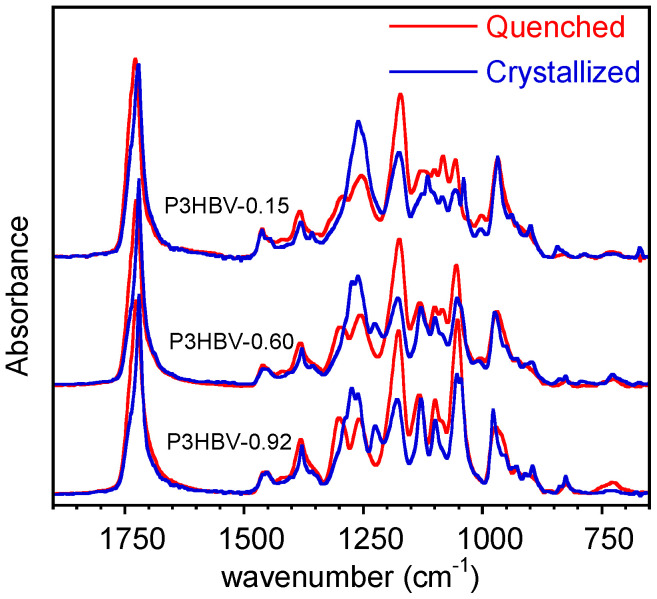
ATR-FTIR spectra of quenched and crystalline P3HBV representative samples at different 3HB molar fractions.

It is well-known that P3HBV copolymers crystallize at low rates, mainly at a high 3HV content [32]. Therefore, the adopted quenching directly on ATR crystals allows for the obtainment of nearly amorphous samples with the exception of P3HB and P3HBV-0.97, as shown later. For the sake of clarity, the spectra of only three representative P3HBV samples with different compositions are reported in Figure 1. The results acquired immediately after the quenching and after 30 min, during which the crystallization process had taken place, are superimposes. For the sake of comparison, all the spectra were normalized with respect of the absorption band at 1453 cm^−1^.

The assignments of vibrational modes of all the absorption bands of PHA spectra are a challenging task, because there are limited systematic studies and a lack of unequivocal spectral interpretation in the literature. A number of papers are focused on deep investigations to the thermally induced phase transition [25,26,27] or to the evaluation of inter-chain hydrogen bond formation [33,34] and are often limited in the analysis of small spectral ranges where C-H (3100–2800 cm^−1^) and C=O stretching (1800–1700 cm^−1^) resonate.

Figure 1 shows that, except for a few bands that are consistent across the samples with varying compositions, the spectra exhibit significant dissimilarity. The main differences are situated in the 1300 cm^−1^–1200 cm^−1^ spectral range, where the bending of CH, CH_2_, and CH_3_ groups resonate, and between 1150 cm^−1^ and 1000 cm^−1^, where the multiple absorption bands are due to C-O-C stretching and C-CH_3_ bending [35,36]. Furthermore, as described in numerous studies reported in the literature, there are notable spectral differences between amorphous and semi-crystalline polymers. These complex changes encompass variations in band intensity, position shifts, and the emergence of new bands. As a result, in a preliminary study, poor correlations and large data scattering were identified between band intensities and the sample composition when comparing as received extracted P3HBV samples, characterized by different and unknown crystallinity.

**Figure 2:**
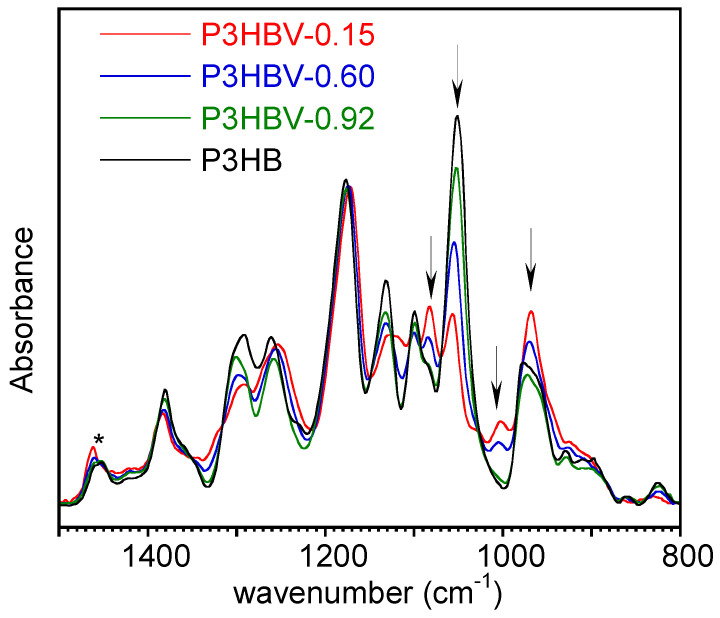
Baseline corrected ATR-FTIR spectra of representative quenched P3HBV and P3HB samples with different 3HB repeating unit molar fractions. The absorbance values were normalized with respect to the intensity of the band at 1453 cm^−1^, marked with an asterisk. The arrows indicate the bands used for the correlation with polymer composition.

In Figure 2, overlaid representative ATR-FTIR spectra of quenched P3HB and of three selected P3HBV samples with varying 3HB content are displayed. They have been normalized with respect to the intensity of the band at 1453 cm^−1^ (δ_as_ CH3 [36]). This absorption has been used as an internal standard in previous studies, as it is not significantly affected by crystallinity [26]. Furthermore, the band intensity is not influenced by nearby absorptions that are sensitive to the composition. Additionally, baseline correction was applied between 1510 cm^−1^ and 848 cm^−1^ wavenumbers, where the spectral characteristics were not influenced by the polymer composition.

Figure 2 shows that the intensity of the absorption bands at 1084 cm^−1^, 1050 cm^−1^, 1004 cm^−1^, and 968 cm^−1^ (marked with arrows) markedly changes as a function of the P3HBV composition. According to the spectra interpretation reported in the literature, the absorption at 1050 cm^−1^ was attributed to C-O symmetric stretching [37] or to C-CH_3_ bending [36], while that at 968 cm^−1^ to C-H bending [38] or C-C stretching [36,39] or to C-O-C stretching [40]. In the samples with high 3HB concentration, the band at 1084 cm^−1^ was observed as a shoulder of the medium intensity band at 1100 cm^−1^, assigned to C-O stretching [40,41] and turn in a resolved band by increasing the 3HV content in the copolymers. The weak absorption at 1004 cm^−1^, located in the valley between the two medium-strong bands at 1050 cm^−1^ and 968 cm^−1^, has never been described in the literature and is not yet attributed to a specific vibration. From a diagnostic perspective, it was considered interesting as it is entirely absent in P3HB homopolymers and evolves from a hump into a well-defined band in P3HBV as the 3HV content increases.

Then, the intensity of these selected absorptions was normalized with respect to that of the band at 1453 cm^−1^ to obtain $R_{\bar{\upsilon }}$ data. The quenched samples with *X_3HB_* =1 and *X_3HB_* = 0.97 showed the presence of an absorption peak at 1225 cm^−1^, either as a well-defined band or as a hump (Figure 2, PHB spectrum). This arises from the partial crystallization [24,25,26] occurring during the transfer of samples from the heating device to the IRE surface or during spectrum acquisition, since P3HB and P3HBV with low 3HV content crystallize rapidly. Consequently, in the subsequent data analysis, samples with *X_3HB_* = 1 and *X_3HB_* = 0.97 were excluded from consideration because of the high scattering of values and associated errors.

**Figure 3:**
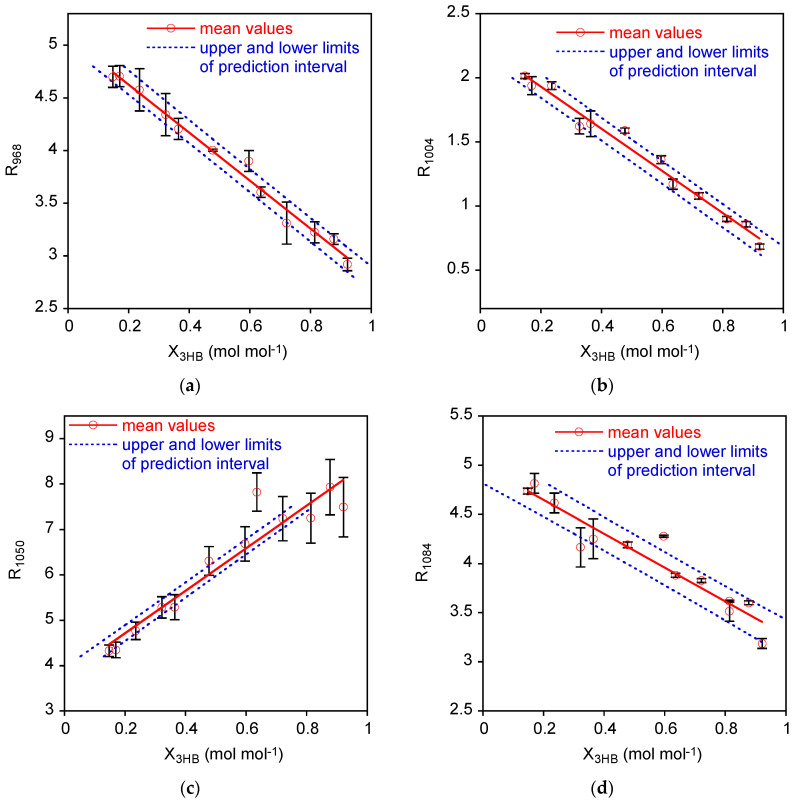
Variation of normalized intensity of the bands at 968 cm^−1^ (**a**), 1004 cm^−1^ (**b**), 1050 cm^−1^ (**c**), and 1084 cm^−1^ (**d**) as a function of 3HB molar fraction. The solid lines are the regressed linear models, and the dotted lines are the lower and upper limits of the prediction intervals.

The mean $R_{\bar{\upsilon }}$ values as a function of the P3HB molar fraction in the 0.15 < *X_3HB_* < 0.92 range are reported in Figure 3a–d with the linear interpolation and the prediction interval on *X_3HB_* described next, in Section 3.2. The error bars correspond to the half-difference between the maximum and minimum values of the data acquired on at least two independent measurements.

### 3.2. Data Analysis

Regarding the parameter estimates and confidence intervals (95%) (Table 1), intercepts present similar relative errors, except for *R*_1050_ with the confidence interval corresponding to 26% variability with respect to the estimated value.

This is due to the great variability of data due to random errors, as evidenced by the high values of *MS_E_* and *MS_PE_* (Table 2).

As far as the slopes are concerned, they are all negative except again for *R*_1050_ data, which present a positive correlation with *X_3HB_* along with high % relative errors (34%), as in the *R*_1084_ case (34%). Both *R*_1050_ and *R*_1084_ regressions present the highest *MS_E_* values, thus determining the highest variation of the confidence band. The high relative errors evidenced by the confidence intervals for *R*_1050_ and *R*_1084_ are also accompanied by the lowest values of the coefficient of determination (*R*^2^), representing the ratio of total variability accounted by the linear model with respect to the total variability of the data. Then, when *MS_E_* is high, the coefficient of determination tends to decrease.

In order to assess the goodness of the linear model, the lack of fit test was performed. The *p* values reported in the table evidenced that, for the *R*_1050_ case, the linear model was not a good approximation of the data, while in all the other cases the data could be adequately represented with linear models (*p* > 0.05).

Then, according to both large confidence intervals on the parameters, low coefficient of determination, and lack of fit tests, the data calculated by using the normalized intensity at 1050 cm^−1^ could not be considered as an optimal choice for the investigated calibration method.

**Figure 4:**
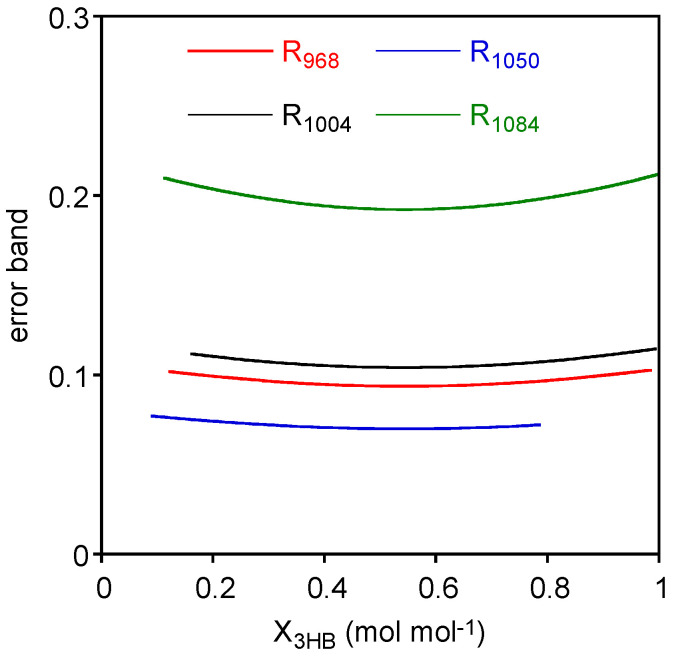
Error bands for the four cases evaluated as the difference between the upper limit and the lower limit of the prediction interval.

Finally, prediction intervals for the *X_3HB_* (*x*) in the measurement phase were estimated considering two replicates of $R_{\bar{\upsilon }}$ (*y*): the prediction intervals on *X_3HB_* were reported in Figure 3 along with experimental data and calibration lines. In order to better visualize the variability of these prediction intervals, the associated error bands have been evaluated as upper *X_3HB_* limit minus lower *X_3HB_* limit and displayed in Figure 4. As expected, the error band for prediction intervals are non-monotonic with a minimum around the mean *X_3HB_* of standard samples.

Considering the comparison among the different cases, we should exclude *R*_1050_ data, as linear calibration is not an adequate representation of data even if this case presents the lowest error band. This can be explained considering the formula for estimating the prediction intervals on *X_3HB_*. The *R*_1050_ case presents the lowest error band, because the high error variability (*MS_E_*) is compensated by the highest slope.

On the other side, *R*_1084_, which presents the second largest error variability, does not benefit from such “compensation” having a low value for the slope. Accordingly, the error bands are the largest ones.

*R*_968_ and *R*_1004_ present similar values of the error bands, and, considering previous statistical analysis of confidence intervals on parameters, coefficient of determination, and the lack of fit tests, these are the best correlations to be considered for assessing the composition of the mixture.

## 4. Discussion

Fourier transform infrared (FTIR) spectroscopy is a widely used technique for the preliminary qualitative characterization of extracted PHAs or, more commonly, for the initial estimation of PHA content in biomasses [42,43]. However, the standard gas chromatography analysis is required to determine the polymer composition, the exact amount of polymer in the biomass, and the purity of the extracted polymer. On the other hand, it can be useful to use a more rapid and facile method to assess the composition of PHAs of unknown origin or to check the extraction procedure using a readily accessible instrument such as FTIR. In an early paper, a method to determine P3HBV composition using FTIR spectroscopy has been proposed [24]. It was based on the evaluation of the integral intensity ratio of the stretching bands of C-H and C=O. The sample preparation consisted of polymer dissolution in chloroform and casting on KBr disks for the spectra acquisition in a transmission mode. In this way, the authors stated that, since all the samples reached the same crystallinity upon solvent evaporation, the relationship between integral intensity ratio and composition of standard samples was linear. Actually, the crystallinity that P3HBV can reach strongly depends on copolymer composition, even in the same sample preparation conditions. This is especially true when a wide composition range is considered.

Then, the key point of the method proposed in this paper is the analysis of quenched amorphous polymer samples. This approach ensures that the initial state of the sample does not influence the measurement, enabling a rapid and straightforward sample preparation and spectrum acquisition with ATR-FTIR. In fact, the ATR device is nowadays very common for its ease of use and is often included into the FTIR apparatus; a heating plate is the only supplementary tool needed. The sample amount needed for the analysis is about 5–10 mg, and, in the adopted instrument set-up, the sample melting, quenching, and spectra acquisition take about 1–2 min. This allows for the rapid building of a custom calibration curve with few standard samples, which is necessary, for example, whenever a different IRE or incident angle of ATR device is used.

The data analysis showed that the normalized band intensity at 968 cm^−1^ and 1004 cm^−1^ are the most reliable to predict P3HBV composition.

## Figures and Tables

**Table 1:** Coefficients (intercept and slope), upper and lower limits for the confidence intervals, and relative errors for the investigated normalized band intensities.

	Intercept	Lower Limit (95%)	Upper Limit (95%)	Relative Error * (%)	Slope	Lower Limit (95%)	Upper Limit (95%)	Relative Error * (%)	*R* ^2^
*R*_968_	5.11	4.95	5.26	6	−2.33	−2.58	−2.07	22	0.930
*R*_1050_	3.77	3.29	4.25	26	4.75	3.95	5.55	34	0.851
*R*_1084_	5.00	4.82	5.18	7	−1.75	−2.05	−1.45	34	0.848
*R*_1004_	2.27	2.18	2.36	8	−1.68	−1.83	−1.53	18	0.953

* The relative error was calculated as the half-difference between the upper and lower limit divided by intercept or slope value.

**Table 2:** Variances related to residual error (*MS_E_*), pure error (*MS_PE_*), lack of fit error (*MS_LOF_*), *F* values for the LOF test, and related *p* values.

	*MS_E_*	*MS_PE_*	*MS_LOF_*	*F* Value	*p* Value
*R*_968_	0.0284	0.0198	0.0423	2.1350	0.0850
*R*_1050_	0.2762	0.1243	0.5191	4.1772	0.0056
*R*_1084_	0.0380	0.0289	0.0527	1.8246	0.1367
*R*_1004_	0.0096	0.0074	0.0131	1.7587	0.1514

## Data Availability

The datasets generated during and/or analyzed during the current study are available from the corresponding author on reasonable request.
